# Minieditorial: Características Clínicas da Hipertensão Arterial Resistente *versus* Refratária em uma População de Hipertensos Afrodescendentes

**DOI:** 10.36660/abc.20200344

**Published:** 2020-07-28

**Authors:** Rui Póvoa

**Affiliations:** 1 Universidade Federal de São Paulo São PauloSP Brasil Universidade Federal de São Paulo (UNIFESP), São Paulo , SP – Brasil

**Keywords:** Hipertensão/complicações, Grupo com Ancestrais do Continente Africano/genética, Estudo Comparativo, Epidemiologia, Infarto do Miocárdio, Acidente Vascular Cerebral

As diferenças do comportamento clínico da hipertensão arterial (HA) nas diversas etnias são, há muito tempo, objetos de intensa investigação científica. As estatísticas da American Heart Association de 2015 verificaram que os afrodescendentes americanos têm a maior prevalência de hipertensão do mundo. Em homens e mulheres negros, não hispânicos, a prevalência ocorreu em 44,9% e 46,1%, respectivamente. ^1^ Embora tais números não sejam universais para todas as populações negras, existem grupos em que os níveis pressóricos são muito mais elevados, como os negros sul-africanos, nos quais a pressão arterial sistólica é 9,7 mmHg mais elevada em comparação aos afro-americanos. ^2 , 3^

As lesões a órgãos-alvo e as complicações são mais prevalentes nos negros em comparação aos brancos ou hispânicos para os mesmos níveis de pressão, além de apresentarem maior resistência ao tratamento. ^4 , 5^

A hipertensão resistente (HR) e a refratária (HRf) acometem uma proporção não desprezível dos pacientes hipertensos. No Brasil, de acordo com o estudo ReHOT, a prevalência é de 11,7% dos hipertensos. ^6^

Ainda se discute muito se a HR e a HRf são fenótipos ou gradações diferentes de uma mesma doença. De modo similar a simples HA que podemos controlar com até três fármacos, a etiopatogenia é multifatorial, e têm importância os fatores genéticos e ambientais. Entre os fatores ambientais, a ingesta de sal e/ou a não excreção salina adequada pelos rins são elementos preponderantes do não controle pressórico. Isso é corroborado no elegante estudo PATWAY-2, que avaliou o quarto fármaco no fluxograma da terapêutica anti-hipertensiva e discriminou a espironolactona como um dos fármacos mais importantes nesse estágio do tratamento. ^7^ Na sequência, o estudo PATWAY-2 Mechanistic encontrou efeitos similares com o cloridrato de amilorida, ambos diuréticos. ^8^

Em vista deste aspecto multifatorial da HA, envolvendo causas genéticas e ambientais, evidenciam-se algumas particularidades, principalmente com relação à gravidade em determinados grupos étnicos. Os indivíduos afrodescendentes, além de terem maior prevalência de HA e consequências mais graves da doença, apresentam maiores lesões a órgãos-alvo e maior morbimortalidade de causa cardiovascular. ^9^ Isso é bem evidente nos afrodescendentes americanos; contudo, em nossa miscigenada população, não temos estudos robustos para averiguar tais diferenças.

Neste trabalho de Macedo et al., ^10^ Características Clínicas da Hipertensão Arterial Resistente *versus* Refratária em uma População de Hipertensos Afrodescendentes, verificou-se nessa população que a HRf é comum, tendo maior prevalência de dislipidemia, história de acidente vascular cerebral (AVC) e maior lesões a órgão-alvo. ^10^ Os achados parecem redundantes e muito similares aos de afrodescendentes americanos, mas refere-se a um achado com muito significado epidemiológico que pode levantar hipóteses para outros estudos que possam decifrar o grande enigma das diferenças da hipertensão em diversos grupos étnicos.

Acredita-se que os afrodescendentes brasileiros sejam diferentes dos americanos e muito parecidos com os negros nativos da África, com exceção da África do Sul. ^2^ Provavelmente, isso tem muito a ver com o tráfico de escravos da África para a América. Sabemos que alguns negros têm, geneticamente, algumas particularidades no sistema renina-angiotensina-aldosterona, com pouca atividade de renina e com uma massa de néfrons menor, e, com isso, menos sódio excretado. Sem conhecer qualquer aspecto fisiopatológico, os escravagistas ingleses lambiam o suor do negro para ver se era salgado ou não, e assim escolher indivíduos preferenciais para o transporte nos porões das naus, sem condições de alimentação e hidratação. ^11 - 13^ Aqueles que conseguiam sobreviver à travessia do Atlântico eram justamente os que apresentavam um sistema de retenção de sódio e água mais eficiente. Assim, no Novo Mundo, com excesso de sal na alimentação, diferentemente da terra natal, evoluíam para uma hipertensão mais grave. Esse caminho do tráfico de pessoas da África para a América do Norte é quase o dobro da distância para o Brasil e, por isso, a seleção dos africanos no Brasil não foi tão acentuada como a americana. No entanto, não existem estudos bem desenhados que possam responder corretamente a tais dúvidas epidemiológicas.

Estima-se que a hereditariedade contribua de 40% a 50% da patogênese da hipertensão, mas pouco se sabe de sua arquitetura genética na identificação de *loci* de genes responsáveis pela elevação pressórica. Os afrodescendentes americanos apresentam índice de renina e aldosterona mais baixo que os brancos para o mesmo nível de ingesta de sódio. ^14^ A sensibilidade ao sal é um fenótipo mais comum em negros, e muito relacionada a resposta pressórica com a variação da ingesta de sódio, mesmo naqueles com baixo índice de renina e aldosterona. ^15^

Desse modo, este trabalho de Macedo et al. ^10^ tem importância na avalição das características próprias das diversas etnias que compõem a população brasileira. O trabalho foi realizado na cidade de Salvador, na Bahia, onde a população de afrodescendentes representa boa parcela dos habitantes. O conhecimento mais profundo de características pressóricas, risco cardiovascular, medicamentos mais efetivos, lesões preferenciais a órgãos-alvo etc. pode levar a planos mais acurados de controle, prevenção e terapêutica. Este estudo de desenho transversal, com avaliação clínica e laboratorial precisa – inclusive com realização de monitoramento ambulatorial da pressão arterial (MAPA) para afastar o efeito do avental branco, situação bastante comum – permitiu conclusões de importância epidemiológica em nossa população afrodescendente.

Será necessário percorrer ainda muitos caminhos para desvendar e encaixar todas as peças do polígono multifatorial de todos os fenótipos da hipertensão, principalmente a HR e a HRf. Ainda não sabemos profundamente qual o papel preciso do sal, do sistema nervoso simpático, do endotélio e de todos os outros fatores relacionados com esta complexa doença chamada hipertensão arterial.
