# Supplementary material for: Minieditorial: Características Clínicas da Hipertensão Arterial Resistente versus Refratária em uma População de Hipertensos Afrodescendentes
Source: Arq Bras Cardiol. 2020 Jul 28;115(1):40–1. [Article in Portuguese] doi: 10.36660/abc.20200344 (PMC8384322; doi:10.36660/abc.20200344)
Supplement: Translation

# Translation: Short Editorial: Clinical Characteristics of Resistant vs. Refractory Hypertension in a Population of Hypertensive Afro-descendants

**Authors:** Rui Póvoa

**Affiliations:** 1 Brazil Universidade Federal de São Paulo (UNIFESP), São Paulo, SP – Brazil

**Keywords:** Hypertension/complications, African Continental Ancestry Group/genetics, Comparative Studies, Epidemiology, Myocardial Infarction, Stroke

Differences in the clinical behavior of arterial hypertension (AH) in diverse ethnic groups have long been the object of intense scientific investigation. The American Heart Association statistics for 2015 found that Afro-descendants have the highest prevalence of hypertension worldwide. In black, non-Hispanic men and women, the prevalence was 44.9% and 46.1%, respectively.^1^ Although such figures are not universal for all Afro-descendant populations, there are groups in which blood pressure levels are much higher, such as South-African blacks, in whom systolic blood pressure is 9.7 mmHg higher when compared to African-Americans.^2 , 3^

Target-organ lesions and complications are more prevalent in Afro-descendants, when compared to Caucasians or Hispanics for the same pressure levels, in addition to the fact that they are more resistant to treatment.^4 , 5^

Resistant (RH) and refractory (RfH) hypertension affect a non-negligible proportion of hypertensive patients. In Brazil, according to the ReHOT study, the prevalence is 11.7% of hypertensive patients.^6^

There is still much debate as to whether RH and RfH are different phenotypes or degrees of the same disease. Similarly to simple AH, which can be controlled with up to three drugs, the etiopathogenesis is multifactorial, and genetic and environmental factors are important. Among the environmental factors, salt intake and / or non-adequate saline excretion by the kidneys are preponderant elements of non-blood pressure control. This is corroborated in the elegant PATWAY-2 study, which evaluated the fourth drug in the antihypertensive therapy flowchart and singled out spironolactone as one of the most important drugs at this stage of treatment.^7^ Subsequently, the PATWAY-2 Mechanistic study found similar effects with amiloride hydrochloride, both diuretic drugs.^8^

Considering this multifactorial aspect of AH, involving genetic and environmental causes, some particularities are evident, mainly regarding disease severity in certain ethnic groups. Afro-descendant individuals, in addition to having a higher prevalence of AH and more severe consequences of the disease, show greater damage to target-organs and greater morbidity and mortality from cardiovascular causes.^9^ This is quite evident in African-American descendants; however, in our mixed population, there are no robust studies to ascertain such differences.

In this study by Macedo et al.,^10^ “Clinical Characteristics of Resistant vs. Refractory Hypertension in a Population of Hypertensive Afro-descendants”, it was found that RfH is common in this population, with a higher prevalence of dyslipidemia, history of stroke and greater damage to the target-organ.^10^ The findings seem redundant and very similar to those of American Afro-descendants, but refer to a finding with a lot of epidemiological significance, which may raise hypotheses for other studies that can solve the great enigma of hypertension differences in different ethnic groups.

Brazilian Afro-descendants are believed to differ from African-Americans and to be very similar to native African blacks, with the exception of South Africa.^2^ This probably has a strong association with the slave trade from Africa to America. It is known that some blacks have, genetically, some peculiarities in the renin-angiotensin-aldosterone system, with little renin activity and a smaller nephron mass, and thus, less sodium is excreted. Without being aware of any pathophysiological aspect, the English slavers tasted the black men’s sweat to verify whether it was salty or not, and thus choose certain individuals for transportation in the ship holds, without food and water conditions.^11 - 13^ Those who managed to survive the crossing of the Atlantic ocean were precisely those with a more efficient sodium and water retention system. Thus, in the New World, with an excess of salt in their diets, unlike in their homelands, they developed a more severe hypertension. This path of human trafficking from Africa to North America is almost twice the distance to Brazil and, therefore, the selection of Africans in Brazil was not as accentuated as the North-American one. However, there are no well-designed studies that can correctly answer such epidemiological questions.

It is estimated that heredity contributes 40% to 50% of the pathogenesis of hypertension, but little is known about its genetic architecture in the identification of the *loci* of genes responsible for high blood pressure. African-Americans have a lower renin and aldosterone index than Caucasians for the same level of sodium intake.^14^ Salt sensitivity is a more common phenotype in blacks, and closely related to the pressure response with the sodium intake variation, even in those with low renin and aldosterone index.^15^

Therefore, this study by Macedo et al.^10^ is important for the assessment of the characteristics of different ethnic groups that comprise the Brazilian population. The study was carried out in the city of Salvador, Bahia, where the Afro-descendant population represents a good portion of the inhabitants. The deeper knowledge of blood pressure characteristics, cardiovascular risk, more effective drugs, preferential target-organ lesions, etc., can lead to more accurate control, prevention and therapeutic plans. This cross-sectional study, with precise clinical and laboratory evaluation – including ambulatory blood pressure monitoring (ABPM) to rule out the white coat effect, a very common situation – allowed conclusions of epidemiological importance in our Afro-descendant population.

Many pathways will need to be traveled to unravel and fit all the parts of the multifactorial polygon of all hypertension phenotypes, mainly RH and RfH. We do not know yet what the precise roles of salt, the sympathetic nervous system, the endothelium and all the other related factors are in this complex disease called arterial hypertension.
